# Plant Microbial Fuel Cells–Based Energy Harvester System for Self-powered IoT Applications

**DOI:** 10.3390/s19061378

**Published:** 2019-03-20

**Authors:** Edith Osorio de la Rosa, Javier Vázquez Castillo, Mario Carmona Campos, Gliserio Romeli Barbosa Pool, Guillermo Becerra Nuñez, Alejandro Castillo Atoche, Jaime Ortegón Aguilar

**Affiliations:** 1 CONACYT, Deparment of Engineering, University of Quintana Roo, Chetumal Q. Roo 77019, Mexico; eosorio@conacyt.mx (E.O.d.l.R.); gbecerra@conacyt.mx (G.B.N.); 2 Deparment of Engineering, University of Quintana Roo, Chetumal Q. Roo 77019, Mexico; 1112480@uqroo.mx (M.C.C.); romeli@uqroo.edu.mx (G.R.B.P.); jortegon@uqroo.edu.mx (J.O.A.); 3 Faculty of Engineering, Autonomous University of Yucatán, Mérida Yucatán 97000, Mexico; acastill@correo.uady.mx

**Keywords:** energy harvesting, internet of things (IoT), plant microbial fuel cell (PMFC), self-powered systems, wireless sensor network

## Abstract

The emergence of modern technologies, such as Wireless Sensor Networks (WSNs), the Internet-of-Things (IoT), and Machine-to-Machine (M2M) communications, involves the use of batteries, which pose a serious environmental risk, with billions of batteries disposed of every year. However, the combination of sensors and wireless communication devices is extremely power-hungry. Energy Harvesting (EH) is fundamental in enabling the use of low-power electronic devices that derive their energy from external sources, such as Microbial Fuel Cells (MFC), solar power, thermal and kinetic energy, among others. Plant Microbial Fuel Cell (PMFC) is a prominent clean energy source and a step towards the development of self-powered systems in indoor and outdoor environments. One of the main challenges with PMFCs is the dynamic power supply, dynamic charging rates and low-energy supply. In this paper, a PMFC-based energy harvester system is proposed for the implementation of autonomous self-powered sensor nodes with IoT and cloud-based service communication protocols. The PMFC design is specifically adapted with the proposed EH circuit for the implementation of IoT-WSN based applications. The PMFC-EH system has a maximum power point at 0.71 V, a current density of 5 mA cm${}^{-2}$, and a power density of 3.5 mW cm${}^{-2}$ with a single plant. Considering a sensor node with a current consumption of 0.35 mA, the PMFC-EH green energy system allows a power autonomy for real-time data processing of IoT-based low-power WSN systems.

## 1. Introduction

Internet-of-Things (IoT)-based wireless sensor networks (WSN) are composed of miniaturized sensor nodes distributed in an area to collect real-time data such as temperature, salinity, water stress, and humidity. Traditional IoT-based WSN systems need to replace their batteries or recharge them when the stored energy is low or exhausted, and the replacement process can be difficult or even impossible. One of the main challenges of this technology is the development of an adequate energy harvesting (EH) system. In this regard, EH systems play a critical role in the fastest growing state-of-the-art electrical applications, such as IoT, smart cities, biosensors, wearable electronics, and autonomous WSNs [1,2,3,4].

In addition, the use of IoT-based and energy harvester systems have been proposed as a solution to diverse engineering problems, such as, cattle and honey bee monitoring [5,6], automated irrigation [7], early pest detection [8] and structural health monitoring [9], among others.

Today, self-powered harvester systems have gathered energy from wind, sun-light and motion, among others [10,11], with the aim of providing uninterrupted energy supplies that can be an attractive and efficient solution for substituting highly polluting batteries of the IoT systems. Likewise, these energy harvester techniques are very cost-effective in consumer electronics and highly scalable in wireless sensor networks. However, ambient-based (e.g., sun-light and wind power) and piezo-motion systems are hardly available in indoor environments.

Plant Microbial Fuel Cells (PMFCs) represent a good fit to gather energy from indoor and outdoor environments. PMFC harnesses the metabolism of micro-organisms as catalysts and uses organic matter to generate electrical energy, reaching power densities of several hundreds of μW cm${}^{-2}$; their main advantage is that they can generate energy from organic matter in the soil as fuel [12,13,14,15,16]. Furthermore, this technology maintains the natural landscape of the place where it is wanted to be implemented; e.g., it can be used as green wall for improving the city environment.

The combination of EH-WSN and Plant Microbial Fuel Cells (PMFC) generates a maintenance-free alternative to environmentally harmful batteries. However, one of the main challenges with PMFC is the dynamic power supply, dynamic charging rates and low-energy supply. Efforts have been made to develop autonomous batteryless sensor nodes through EH and the implementation of MFCs [14,17,18,19].

For example, a MFC power supply is presented in [17] for low-power temperature monitoring. In the design, a power management unit is proposed for low input voltage operation showing the use of MFC-based renewable energy under a realistic operation. [18] presents an interesting floating MFC used as energy harvester for signal transmission from natural bodies of water. The design proposes a power management system based on a step-up DC/DC converter and a low-power data transmission system with Sigfox technology, while [14] employs an intelligent energy harvesting scheme for MFCs. The maximum power point tracking and voltage overshoot avoidance algorithms are implemented with the aim of providing better performances in the MFC.

In [19], a review of the effects of plants on PMFC, the role of microbes driven by soil physiochemical and biological characteristics, and engineering aspects involved in designing efficient configurations are presented.

Although considerable progress has already been achieve on the implementation of low-cost and green energy systems to monitor real time data, there still remain some relevant problems to solve. One of the major constraints for PMFC-EH is operating at low input voltage and at very low power completely autonomously and without a battery. For IoT-based WSN applications, the natural ambient fuel is assumed to be unlimited, and therefore, the priority is to harvest sufficient electrical energy regardless of the fuel consumption rate.

In this paper, a PMFC-EH sensor node system is proposed for implementing a self-powered design for IoT applications. The design is composed of a nano-power boost converter adapted to a PMFC design, a Dynamic Power Management (DPM) for low-power consumption, and an ultra-low power microcontroller. The PMFC is specifically designed for the sustainable operation of the proposed IoT-based wireless sensor node. The selection of the Cordyline fruticosa plant, typical of the south east region of Mexico, the mix of soil components, PMFC dimension, fertilizer, and the Cu/Zn electrodes, are capable to generate a stable and continuous power supply for the proposed system.

The PMFC-EH sensor node analysis can be easily accessed through a friendly online user interface for the measurements of temperature and humidity sensors. A dynamic power management strategy is also adopted to harvest the maximum energy from a PMFC to provide a self-autonomous operation of the wireless sensor node.

The rest of the paper is organized as follows: Section 2 details the design and PMFC characteristics. The implementation of the PMFC-EH is described in Section 3. The IoT sensor node prototype, DPM strategy and the measurement data analysis are also reported. Section 4 presents the experimental results of the system and the software interface. Concluding remarks are given in Section 5.

## 2. Plant Microbial Fuel Cell: Materials, Methods and Calculations

**Figure 1:**
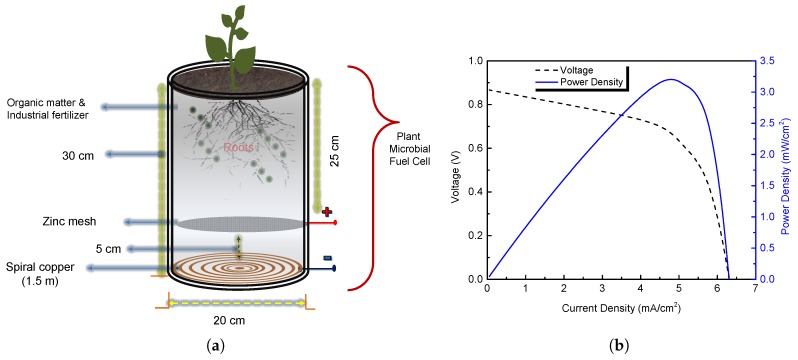
(**a**) Schematic representation of the proposed PMFC in the set of experiments: PMFC used with mix soil, fertilizer and the Cordyline Fruticosa plant; (**b**) Typical performance of a PMFC (*I*-*V* and *P*-*V* characteristic curves).

PMFC is a renewable and clean bioengineering technology based on two principles: (i) rhizodeposition, or loss of organic compounds in plant roots; and (ii) electrochemical electricity generation by active bacteria via dead root degradation. Currently, researchers are focused on increasing the power output of the PMFC; however, the electrical parameters depend on many factors, such as substrate availability per square meter, plant growth area, distance between the anode and cathode, type of materials, and the local mix soil factors: humidity, electrical conductivity, temperature, and pH, among others [20,21,22,23,24,25,26,27]. Therefore, it is necessary to analyze the components in order to design a PMFC to deliver the maximum power density as can be seen in Figure 1. PMFCs are a specific form of Microbial Solar Cells; systems in which Microbial Fuel Cells or Microbial Electrolysis Cells depend from sunlight [28,29,30].

Figure 1a illustrates the proposed PMFC design with a mix of soil components, fertilizers, and the Cordyline fruticosa plant typical of the southeastern region of México. This plant of the monocots family has the capacity of providing a C4 carbon fixation which increase the rhizosphere surface area for microbiome proliferation [31]. The plant produces organic matter from sunlight and CO${}_{2}$ via photosynthesis. Up to 70% of this organic matter ends up in the soil as dead root material, lysates, mucilage and exudates. This organic matter is oxidized by bacteria living at and around the roots, releasing CO${}_{2}$, protons and electrons.

The PMFC is implemented with two 14 caliber electrodes, and has a transversal area of 0.021 cm${}^{2}$ to measure electrical power generation. A spiral copper cathode is placed at the bottom of the PMFC at a depth of 30 cm from ground level, and a Zinc mesh positioned 5 cm above the cathode serves as an anode. Both electrodes have a circular area and a diameter of 20 cm. The anode is coupled, via an external load to a cathode, and the protons that were released at the anode side travel through a membrane or spacer towards the cathode [32,33,34,35].

The electrical performance of the PMFC is assessed with a Metrohm Autolab potentiostat. Note that the maximum power extracted from a PMFC depends heavily on several parameters, such as microbial populations, electrode contacts, electrochemical parameters, cell temperature, and the external load resistance ($R_{ext}$). Power density ($P_{D}$) represents the amount of energy that the PMFC system can deliver based on its volume. This rate of energy, in Watts per square centimeters (W cm${}^{-2}$), is expressed as follows:

(1) $$P_{D}=\frac{V_{cell}^{2}}{A_{anode}\cdot R_{ext}},$$

where $V_{cell}$ is the measured cell potential in volts (V), the external load resistance $R_{ext}$ is expressed in ohms (Ω), and $A_{anode}$ represents the area of anode.

On the other hand, Figure 1b illustrates the current-voltage (*I*-*V*) curve of a typical PMFC; from the analysis of the figure, it can be deduced that the maximum power point occurs at the optimal voltage ($V_{OP}$) and optimal current density ($J_{OP}$), which represents the ideal PMFC operation point. PMFC performance is determined by analyzing cell potential, the current generation and the internal resistance. Any PMFC source has an internal resistance ($R_{int}$), and the maximum power is transfered to the load when $R_{int}$ is equal to the external resistance $R_{ext}$ [21,22]. The internal resistance is calculated as follows:

(2) $$R_{int}=\frac{V_{ocv}-V_{cell}}{i_{D}},$$

where $V_{ocv}$ and $i_{D}$ represent the open circuit voltage and the circuit current (A) of the PMFC, respectively. In this study, the PMFC power density analysis was carried out under natural light conditions with an average light intensity of 900 W m${}^{-2}$ for 12 h per day. Solar irradiation data for the period of study were obtained using a weather station located at The Center for Research and Advanced Studies of the National Polytechnic Institute (Centro de Invesigación y de Estudios Avanzados del Instituto Politécnico Nacional), in Mérida Yucatán, México (21.02132 N, −89.62685 W).

## 3. PMFC-Energy Harvesting Sensor Node

Novel IoT-based wireless sensor networks face the challenge of surpassing the limited lifespan associated with batteries. To ensure an autonomous energy operation of the sensor node, the potential required for sensing, processing, and communicating must be balanced with the energy harvested from the proposed PMFC.

**Figure 2:**
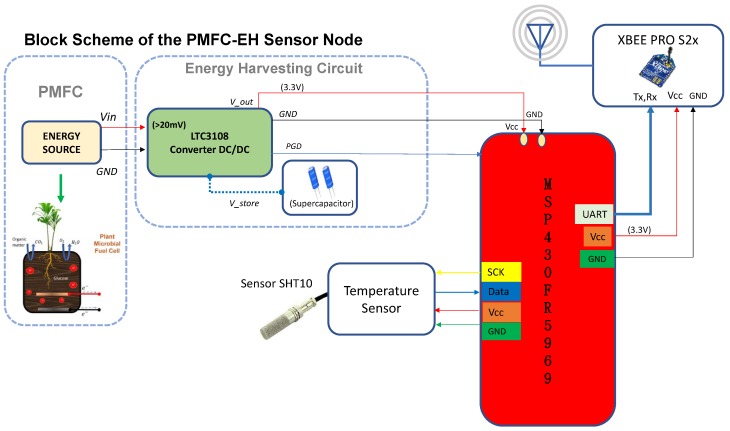
Conceptual PMFC-EH system for the IoT applications proposed in this paper.

This section describes the architecture of a PMFC-EH sensor node for IoT applications. Figure 2 shows the block diagram of the proposed electronic system for the sensor node. The system is composed of the PMFC, an energy harvester circuit, a sensor node, a microcontroller unit (MCU), and a wireless transmission module. In addition, it is necessary to implement an energy strategy to configure the PMFC-EH system for ultra-low power consumption with IoT services and cloud storage management.

### 3.1. Energy Harvesting Circuit

**Figure 3:**
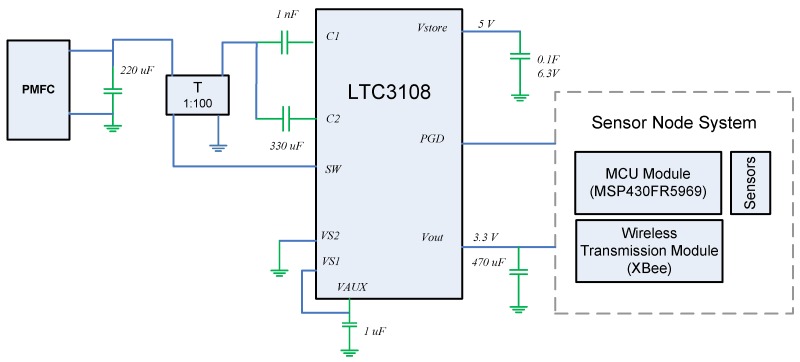
EH subsystem based on LTC3108 power management circuit.

Figure 3 shows the main components of the EH subsystem. The circuit contains a DC/DC converter that manages energy from an input voltage as low as 20 mV. The LTC3108 has a typical current consumption of less than 6μA and uses a MOSFET switch to track and extract the maximum power from the PMFC, inspite the variations in power supply and dynamic charging rates. This allows it to boost input voltages as low as 20 mV-high enough to provide multiple regulated output voltages to power the IoT-based sensor node. The oscillation frequency is determined by the inductance of the secondary winding transformer and is typically in the range of 10 to 100 kHz. According to Figure 2 and Figure 3, as soon as the pin $V_{out}$ exceeds 3.3 V, $V_{store}$ is allowed to charge up the supercapacitors that depend on the input voltage and transformer turns ratio; however, the current is limited to 4.5 mA. The power management circuit is configured with VS1 tied to VAUX and VS2 to GND, to deliver a regulated $V_{out}$ of 3.3 V to the sensor node.

LTC3108 circuit was designed to accumulate and manage energy over long period of time. In this case, the PMFC energy is accumulated in supercapacitors and then, the PMFC-EH system is able to power the MSP430 microcontroller and components. Note that another efficient power management strategies have been implemented using the LTC3108 [36,37,38]; however those configurations were not considered for implementing the proposed PMFC-EH system.

### 3.2. Sensor Node System

**Figure 4:**
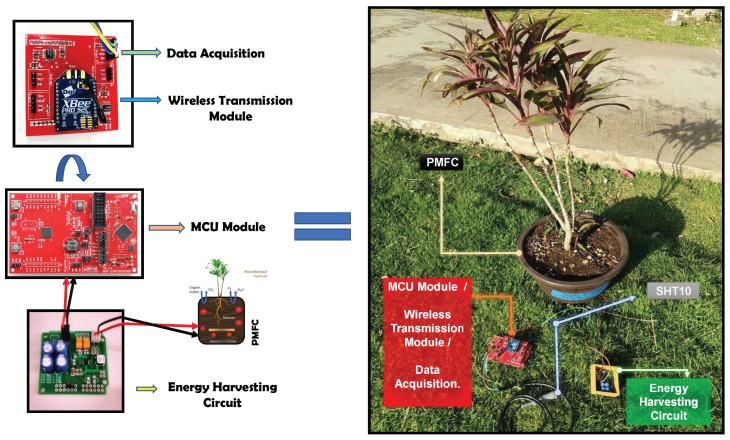
PMFC-EH Sensor Node.

An ultra-low power sensor node architecture has been designed to perform temperature measurements in real time. The EH subsystem is adapted to the PMFC and provides the required energy to the sensor node. Figure 4 illustrates the integration of the ultra-low power sensor node design in a test field. This architecture is composed of three sections: (a) data acquisition and signal conditioning; (b) MCU; and (c) wireless transmission:

(a) Data acquisition and signal conditioning module: The temperature data is acquired through the SHT10 sensor IC [39]. The SHT10 delivers a 12-bit digital output precission with a typical accuracy of 0.5 ${}^{\circ }$C. It can be powered from a voltage range of 2.4 V to 5.5 V, typically consuming 0.9 mA when in operation and a maximum of 1.5μA in sleep mode. The serial interface of the SHT10 is optimized for sensor readout through the I2C communication protocol.

(b) MCU module: The selected MCU is an ultra-low power microcontroller from Texas Instruments with embedded Ferroelectric Random Access Memory (FRAM) technology. The MSP430FR5969 MCU device features 64KB of embedded FRAM, a nonvolatile memory, high endurance, and high-speed write access. Note that the MCU supports CPU speeds of up to 16 MHz and has integrated peripherals, such as ADC and timers. The MCU also supports a wide range of voltages (1.8 V to 3.6 V) and its 16-bit RISC architecture includes the standby (LPM3) and sleep (LPM3.5) low-power modes, which have been optimized to achieve extended battery life in energy-limited applications.

(c) Wireless transmission module: The communication protocol for the sensor node is the IEEE 802.15.4 WPAN Zigbee. The XBee PRO S2C radio modem module is a low-cost, low-power data transceiver unit for WSN and IoT applications. This module provide wireless connectivity to end-point devices and operates with a power-down current below than 1μA. The transmission power is 18 dBm, which reaches distances of up to 1 mile (i.e., line-of- sight range) at a maximum data rate of 250 kbps.

The XBee module is adapted to the MSP430FR5969 MCU host device via a serial port. The XBee communicates the data with an UART protocol, through the serial pins of the MCU, as illustrated in Figure 2. ZigBee is used in WSN, and each node is configured as an end-device in a star topology. The data information over the network is transmitted to the gateway, which collects sensor data, and uploads the information to the cloud.

### 3.3. Strategy for the Ultra-Low Sensor Node Power Consumption

Dynamic Power Management (DPM) is a technique used to manage the power and performance of IoT-based wireless sensor networks [40]. DPM reduces energy usage by dynamically controlling component switching in low-power modes [41]. A Scheduled Switching Mode (SSM) manages the state transitions, allowing power savings for extending sensor node’s life.

**Figure 5:**
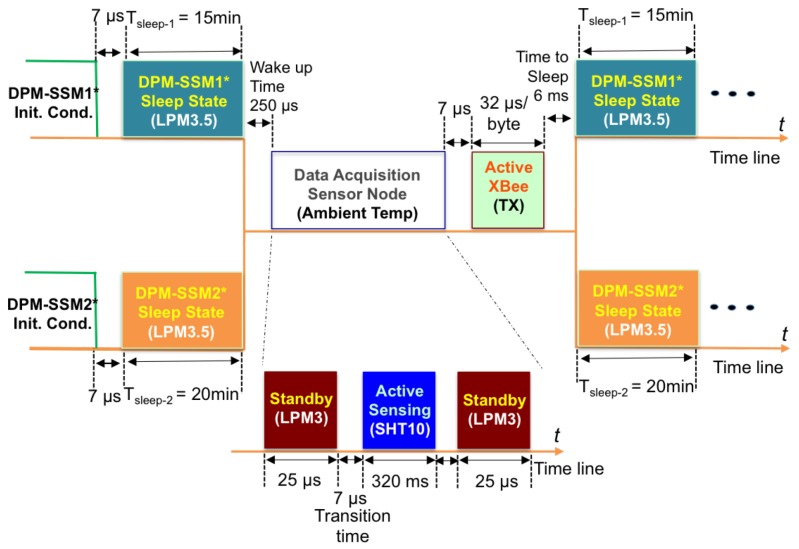
Dynamic Power Management Strategy for Ultra-Low Power Consumption.

Figure 5 shows the SSM stages from data acquisition to wireless transmission. Depending on the type of DPM-SSM stage, the node can consume different levels of battery capacity; i.e., switching from Standby to Sleep mode takes 6 ms and operates with a power down current of 65μA. Transition times between states are also considered in the DPM model, with all values taken from device datasheets.

In this study, two DPM-SSM stages are implemented, based on the PMFC-EH capacity and the quasi-static behavior of data temperature during the day. The strategy dynamically switches from DPM-SSM1 (from 6:01 a.m. to 7:00 p.m.) to DPM-SSM2 (from 7:01 p.m. to 6:00 a.m.) with sleeping periods of 15 and 20 min, respectively. According to Figure 5, once the sensor node system is in Wake-up with a transition time of 250μs, the Standby and the Active-Sensing nodes are sequentially activated for a period of 320.03 ms, with an average current consumption of 5.06 mA. The Active XBee is the state that consumes the most energy, with a typical current of up to 49.09 mA. Therefore, both DPM-SSM stages adopt switching to the Sleep state from the Active XBee state. These DPM-SSM strategies are carried out in order to take full advantage of the PMFC-based battery recovery effect.

### 3.4. Cloud Storage and IoT Services

The Zigbee communications protocol is connected to a local gateway and then to the Internet, uploading the measured data to the cloud. The protocols, Message Queue Telemetry Transport (MQTT), and Representational State Transfer (REST) are used to provide low-latency, small-packet sizes and a stable communication for resource constrained devices. The bandwidth requirements are extremely low, and the nature of the protocol makes it very energy efficient [42]. REST is an architectural style that offers desirable properties, such as performance, scalability, and modifiability, enabling services to work on the Web. A service based on REST is called a RESTful service oriented data networking. The IoT-based systems provide cloud-based data storage and analysis services. When being powered-on or reset, the sensor node requests data from the attached devices every 15 or 20 min. In local communication between the XBee radio modems (over IEEE 802.15.4), the XBee-PRO S2 devices are configured to transmit data, and MQTT transfers the data in the form of messages from the sensor node to a gateway or server (broker). The Publish/Subscribe model used in MQTT is mapped to resource observers. PUT and GET operations on HTTP/REST are also integrated into the MQTT broker.

## 4. Results and Discussion

In this section, the design results of the PMFC for IoT applications are analyzed.

### 4.1. PMFC Comparative Analysis

**Figure 6:**
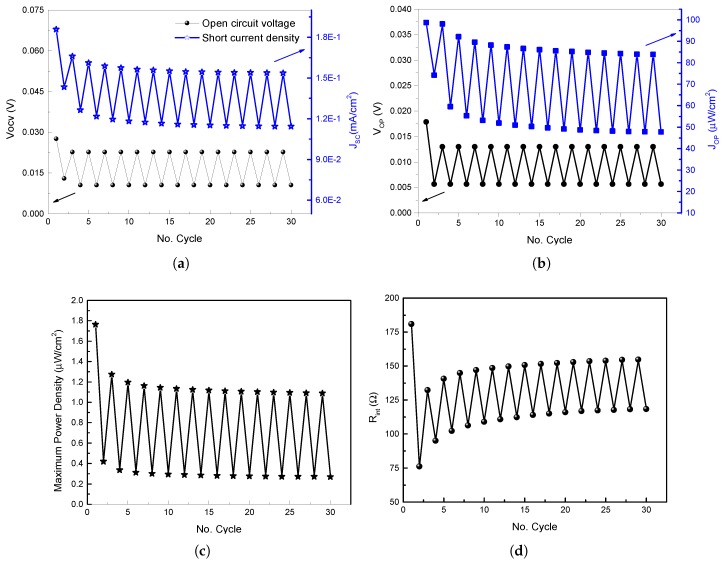
PMFC stainless steel membrane performance comparative: (**a**) open circuit voltage vs short current density; (**b**) optimal open voltage vs optimal current density; (**c**) maximum power density behavior; and (**d**) internal resistance behavior.

In order to guarantee the correct use of the PMFCs in low power IoT applications, several PMFC were designed for this aim. In this sense, in our conducted experiments was observed that PMFCs with Cu/Zn electrodes provide a better performance in comparison to PMFCs designed with traditional stainless steel electrodes. Figure 6 shows the performance of the PMFC designed with the characteristics presented in Section 2 with the incorporation of stainless steel electrodes. As can be seen, the average open circuit voltage $V_{ocv}$ for this PMFC is 15 mV, the short-circuit current $I_{sc}$ is 0.12 mA. Likewise, the maximum voltage $V_{max}$, maximum current $I_{max}$, maximum power $P_{max}$, and the maximum internal resistance $R_{int}$ can be observed. The time cycle period for this experiment was fixed to 20 min for a total test duration equal to 600 min.

**Figure 7:**
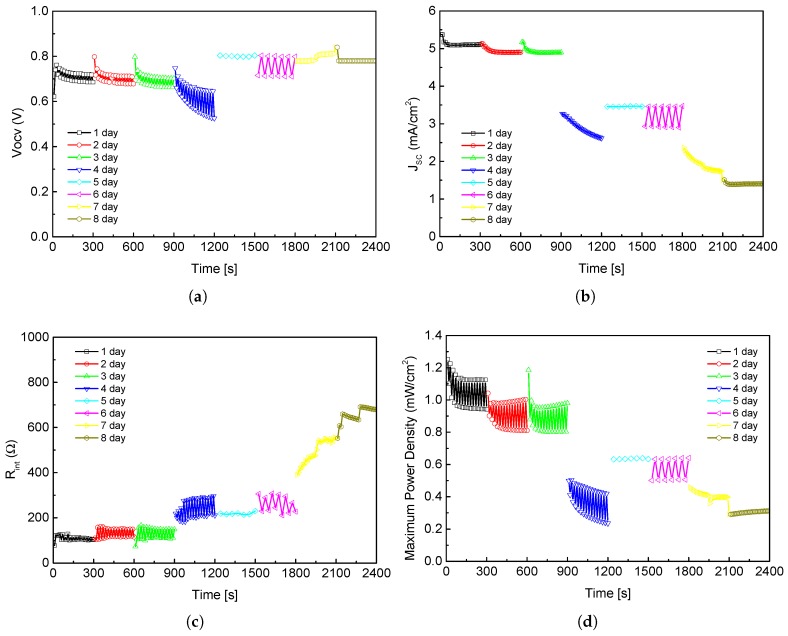
PMFC Cu/Zn performance comparative: (**a**) open circuit voltage; (**b**) short current density; (**c**) internal resistance; and (**d**) maximum power density.

On the other hand, the PMFC was also analyzed with Cu/Zn electrodes as illustrated in Figure 7. As can be seen, the electrical parameters were increased in several order of magnitude, which make possible the PMFC use for low-power consumption applications. Likewise, in order to show the stability of the PMFCs, Figure 7 presents the PMFC behavior during one week. For this experiment nor water neither fertilizers were added with the aim of testing the PMFC response under severe conditions. The samples were acquired at the same hour of day (12:00 p.m., from 10 to 17 December 2018) along the week. A total of 240 samples were acquired in a period of time of 2400 s; i.e., 30 samples per day with a sample period of 10 s.

**Figure 8:**
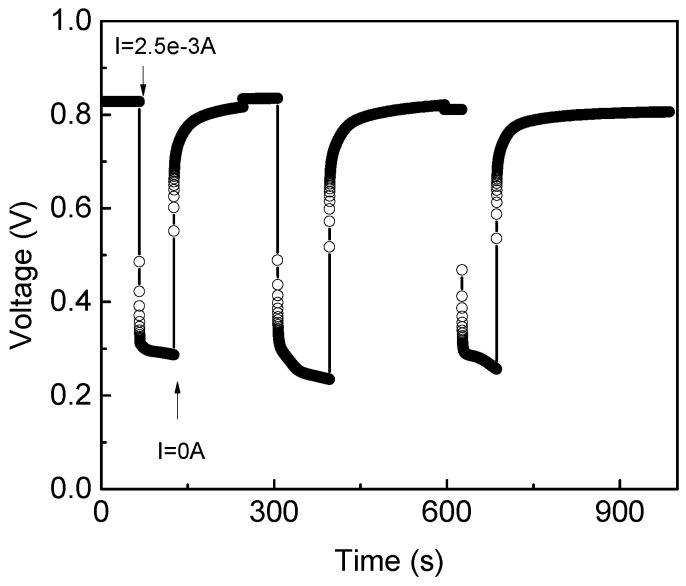
PMFC behavior under charge and discharge.

In addition, the PMFC was stressed during a week using a resistive load equal to 320 Ω. Figure 8 shows the behavior of the PMFC under charge and discharge of its electrical potential. As can be seen, the electrical potential decrease up to 0.25 V for providing an average current equal to 2.5 mA.

After the analysis, it is concluded that this PMFC can be used for WSN-IoT applications where the PMFC is part of the natural environment; i.e., wireless sensor nodes (WSN) for smart farming applications or agriculture 4.0; WSN energized by a PMFC where the plant is a green wall or a green roof. In all cases, the PMFC electrical potential would be improved via water and fertilizer irrigation as can be seen in next subsection. In the test case experiment, the electrodes have not been suffered corrosion after four months.

### 4.2. PMFC Design for Self-Powered WSN-IoT Applications

The PMFC design experiment sets up an IoT scenario for evaluating the performance considering a load resistance of $R_{ext}=120\;\Omega$. The experiment deals with the addition of a mix of organic materials in the form of an industrial fertilizer known as FERTIQUIM® at 11:1 (wt/wt) in dry weight and 100% humidity [43]. This fertilizer ensures the presence of electrochemically active bacteria in the PMFC experiment based on a chemical composition of: 18% Nitrogen-*N*, 18% Ammonium-$NH_{4}$, 46% Phosphorus pentoxide P${}_{2}$O${}_{5}$, 2.20% Soluble Sulfur *S*, and 10% pH in solution (level 6–7).

**Figure 9:**
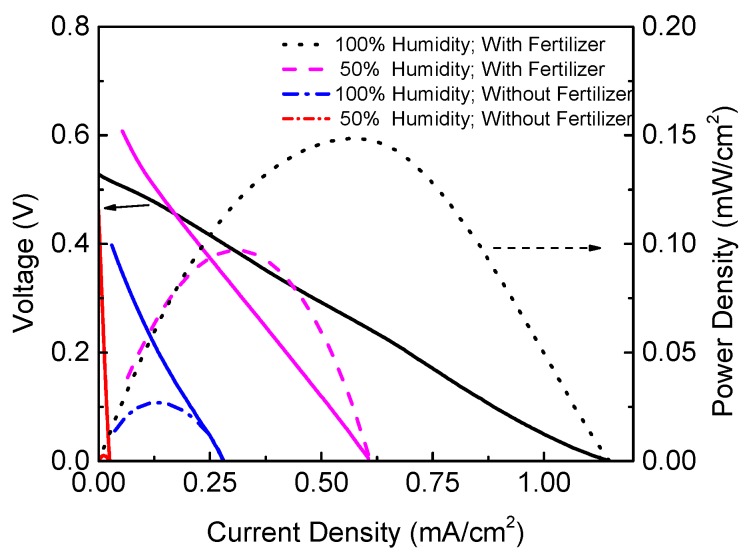
Fertilizer PMFC comparative.

Figure 9 shows the influence of fertilizer in the PMFC. The experiment tests the PMFC behavior with and without the FERTIQUIM fertilizer. Note that the power, current and voltage in the polarization curve is improved in the experiment.

**Figure 10:**
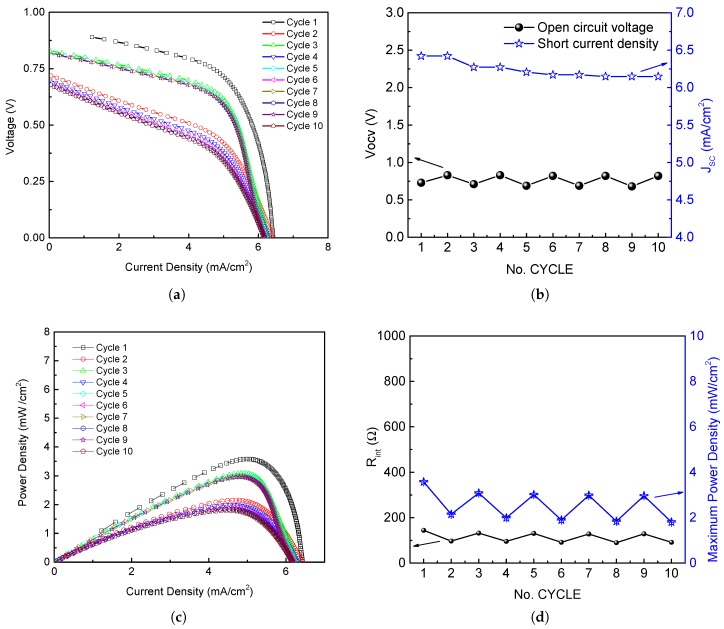
(**a**) Polarization curve of PMFC when the controlled potential is maintained for ten cycles; (**b**) Comparative analysis of the open-circuit voltage (black line) and short-circuit current (blue line) for ten cycles; (**c**) Power curve of PMFC; and (**d**) maximum power density (black curve) per cycle and internal resistance parameter (blue curve) per cycle.

The potential required by the sensor node for processing, sensing, and communicating is balanced with the energy harvested from the PMFC. In this regard, the power energy generated by the PMFC and the stability of the PMFC electrical parameters, such as open circuit voltage $V_{ocv}$, short-circuit current $J_{sc}$, maximum power $P_{max}$, and $R_{int}$, were analyzed (see Figure 10).

Figure 10a shows the polarization curve of the PMFC under stress. The current density is nearly constant at low voltage, however, it presents variations at high rates. This hysteresis in the polarization curve is related to the initial stabilization of electrons and protons produced by bacteria [44].

The $V_{ocv}$ (black line) and $J_{sc}$ (blue line) of the PMFC are illustrated in Figure 10b for time cycle periods of 20 min, according to the DPM-SSM strategy presented in Section 3.3. In the results, $J_{sc}$ tends to decrease smoothly producing variations of up to 0.5 mA cm${}^{-2}$ across the cycles. $V_{ocv}$ has variations of 0.7 V–0.8 V at the same number of cycles.

Figure 10c shows different $P_{D}$ curves. The $P_{D}$ reaches a maximum value of 3.5 mW cm${}^{-2}$, corresponding to a $J_{OP}$ equal to 5 mA cm${}^{-2}$ and a $V_{OP}$ of 0.7 V. The figure analysis shows that the hysteresis is accentuated around the $P_{max}$ density point with variations of up to 1.5 mW cm${}^{-2}$. According to the maximum power transfer theorem, maximum power is achieved when $R_{ext}$ is equal to $R_{int}$ [23]. Thus, $R_{int}$ is calculated per cycle using (2), with results that oscillate between 90 Ω to 150 Ω. Figure 10d presents the results of the maximum power density and the PMFC internal resistance.

The maximum PMFC power transfer has also been discussed in [45,46]. Song et al. in [45] developed a sediment microbial fuel cell (SMFC) with lower external resistances, resulting in higher anode potentials. Lyon et al. [46] monitored a power density production with a MFC for 18 days, varying the external resistance, finding that during the initial period of higher maximum power output, two peaks appear on the polarization curve. These peaks are related to separate populations of bacteria that produce electricity. Song et al. [45] observed that power density is improved with increased external resistance, achieving a power density of 3.15 mW cm${}^{-2}$.

### 4.3. Sensor Node Power Consumption: DPM Strategy

**Figure 11:**
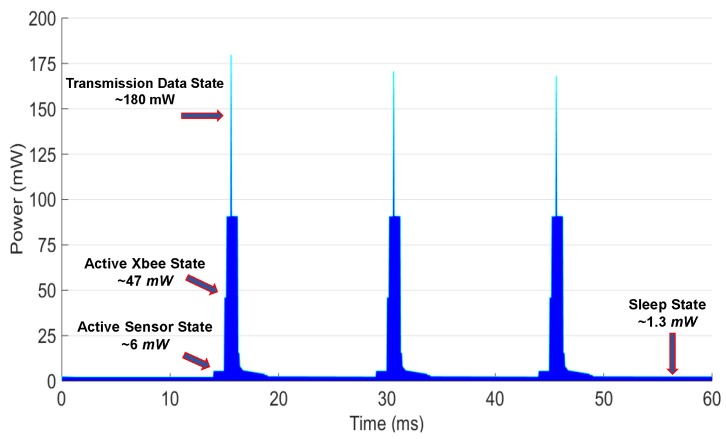
DPM SSM1 strategy.

In order to test the power consumption of the sensor node, the data acquisition, MCU, and transmission states are verified to analyze the energy consumed by data transmission from the sensor nodes to a gateway. The nodes are configured according the DPM strategy of Section 3.3. Real-time measurements of sensor node energy consumption in each operating state were performed. Figure 11 shows the SSM1 stage of the DPM strategy for the PMFC-based EH system, and illustrates the sequence of states over time. After sleeping for 15 min ($T_{Sleep}$ = 900 s), the system is woken up, and the sensing state is activated in the sensor module for a period of $T_{Sensing}=$ 1 ms. Next, the XBee state is activated for $T_{ActiveXbee}=$ 0.5 ms. Finally, the XBee transmission data state requires $T_{Xbee_{TX}}=$ 2 ms.

Table 1 summarizes the measured power consumption results for all configuration states, corresponding to the dynamic behavior of the DPM SSM1 presented in Figure 11 and DPM SSM2.

To estimate the power consumption of the sensor node, the EnergyTrace software was used for real-time energy/power measurements designed specifically for ultra-low-power applications. EnergyTrace technology is included in Code Composer Studio version 6.0 and newer [47]. The resulting mean power consumption value is equal to 1.29 mW and 1.27 mW for the DPM SSM1 and SSM2, respectively.

The EnergyTrace yields information about energy consumption of the internal state of the microcontroller. These states include the ON/OFF status of the peripherals and all system clocks as well as the low power mode (LPM) currently in use.

### 4.4. IoT Application

**Figure 12:**
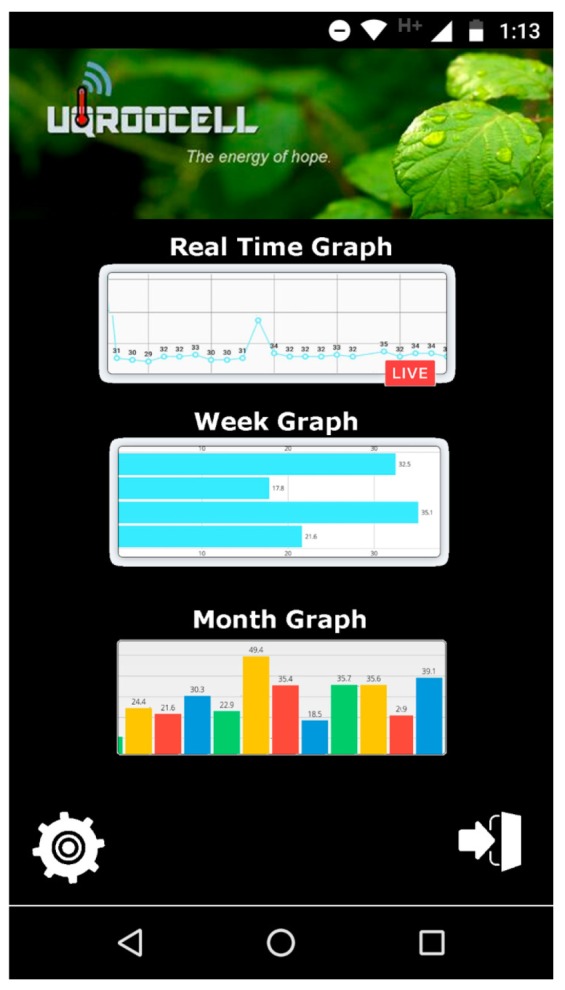
IoT Application: web-based supervision system.

A self-powered PMFC-based energy harvester system for WSN-IoT applications has been implemented. The system is capable of performing temperature data acquisition, cloud-based data storage services, and analysis, as a proof of concept. Likewise, a web-based monitoring system has been developed for online presentation of the measured data. Figure 12 shows the main menu of the user interface on an Android mobile device. The main menu is composed of three main icons: the Real Time Graph, which opens a sub-window where the acquired temperature data are displayed in real-time over a period of time; the Week Graph, which displays the temperature mean by week via a new sub-window; and the icon Month Graph, which shows the mean temperature of the previous months in a new sub-window. The data are also stored in the cloud and are available for analysis via other statistical software applications.

### 4.5. Discussion

The effectiveness is one of the main concerns in the design of PMFC as power sources for IoT applications in indoor and outdoor scenarios. The problem is to estimate the bio-reactions in the PMFC due the soil composition, the electrode materials, type of plant, fertilizers, among others, that impacts in the PMFC performance. For example, the oxygen concentration of the cathode is subject to limitations in oxygen diffusion into the electrode. Also, in [16] it is described that the humidity level in the electrode needs to be high in order to transport protons. In [19,48] different bio-reactions are generated according to the electrode material affecting the PMFC output power. Literature suggest that plants also play a key role in PMFC design. Moreover, an study of biomass production with different plants in PMFCs is shown in [49].

In this study, an energy harvester (EH) circuit is adapted to extract the maximum power from the PMFC, even when the internal impedance of the system, the output power and the changing rates are time-variant. The EH LTC3108 circuit is able to boost input voltage as low as 20 mV, high enough to provide a regulated output of 3.3 V for powering the MSP430FR5969, the XBee Pro S2C radio modem and sensors. Our proposed PMFC generates a stable output voltage of 0.7 V (in average) and a current flow of 5 mA.

The autonomous operation of the sensor node is achieved with a power consumption of only 1.27 mW (mean value) and a PMFC generation of up to 3.5 mW cm${}^{-2}$. The test case scenarios indicate that the temperature data transmission rate using the DPM SSM1 and SSM2 is very well supported. Figure 10 illustrates the energy-efficient operation of the sensor node with a PMFC design based on a local plant and organic soil matter from southeastern México. However, new strategies can be proposed to improve the PMFC effectiveness in future studies.

## 5. Conclusions

This paper proposes a self-powered PMFC-based harvesting system for IoT applications. This solution presents an affordable design for implementing sensor nodes under the paradigm of batteryless and EH systems. A complete performance-study related to the design of the PMFC was presented, and a new PMFC-EH system was introduced and tested. Likewise, a comparison between different PMFCs was performed considering stainless and Cu/Zn electrodes. In addition, the Cu/Zn PMFC was evaluated under different humidity and fertilizer conditions. In order to corroborate the use of the proposed PMFC-EH system in IoT applications, the PMFC was stressed along a week showing a good performance and stability to be used in self-powered circuit designs.

The PMFC-EH system was also configured with a DPM strategy, which allows managing the stored energy in a super capacitor unit. Likewise, a web-based temperature interface was designed, which shows the versatility of the proposal for working under the IoT application concept. The designed PMFC is able to provide 3.5 mW cm${}^{-2}$ with 0.7 V and 5 mA. According to the field test, the PMFC-EH is able to generate enough energy for an autonomous batteryless operation of the sensor node.

Finally, the release of ultra low power devices makes possible to think in the integration of low-power wireless sensor networks into the Internet of Things applications. Thus, this paper deals with use of alternative energy sources based on PMFCs instead of traditional techniques based on PV cells, piezoelectric, thermoelectric, among others. In this sense, PMFCs come to increase the range of possibilities for implementing EH techniques in wireless sensor networks.

## Figures and Tables

**Table 1:** Power Consumption results of the PMFC-based EH system for IoT applications.

	DPM SSM2	DPM SSM1
System		
Time	2523 s	1862 s
Energy	3266 mJ	2375 mJ
Power		
Mean	1.29 mW	1.27 mW
Min	0.0 mW	0.00 mW
Max	178.8 mW	179.74 mW
Voltage		
Mean	3.58 V	3.58 V
Current		
Mean	0.36 mA	0.35 mA
Min	0.0 mA	0.00 mA
Max	49.90 mA	50.12 mA

## Abbreviations

The following abbreviations are used in this manuscript:

SSM	Scheduled Switching Mode
WSN	Wireless Sensor Networks
IoT	Internet-of-Things
EH	Energy Harvesting
PMFC	Plant Microbial Full Cells
DC	Direct Current
MCU	Microcontroller Unit
DPM	Dinamic Power Management
LPM	Low Power Mode
REST	Representational State Transfer
MQTT	Message Queue Telemetry Transport
